# Evolutionary specialization of MscCG, an MscS-like mechanosensitive channel, in amino acid transport in *Corynebacterium glutamicum*

**DOI:** 10.1038/s41598-018-31219-6

**Published:** 2018-08-27

**Authors:** Yoshitaka Nakayama, Kosuke Komazawa, Navid Bavi, Ken-ichi Hashimoto, Hisashi Kawasaki, Boris Martinac

**Affiliations:** 1 Molecular Cardiology and Biophysics Division, Victor Chang Cardiac Research Institute, Darlinghurst, NSW 2010 Australia; 2 Department of Green and Sustainable Chemistry, Tokyo Denki University, 5 Asahi-cho, Senju, Adachi-ku, Tokyo, 120-8551 Japan; 3 St Vincent’s Clinical School, Faculty of Medicine, The University of New South Wales, Darlinghurst, NSW 2010 Australia; 4 Present Address: Institute for Biophysical Dynamics, University of Chicago, Chicago, IL 60637 USA

## Abstract

MscCG, a mechanosensitive channel of *Corynebacterium glutamicum* provides a major export mechanism for glutamate in this Gram-positive bacterium, which has for many years been used for industrial production of glutamate and other amino acids. The functional characterization of MscCG is therefore, of great significance to understand its conductive properties for different amino acids. Here we report the first successful giant spheroplast preparation of *C. glutamicum* amenable to the patch clamp technique, which enabled us to investigate mechanosensitive channel activities of MscCG in the native membrane of this bacterium. Single channel recordings from these spheroplasts revealed the presence of three types of mechanosensitive channels, MscCG, MscCG2, and CgMscL, which differ largely from each other in their conductance and mechanosensitivity. MscCG has a relatively small conductance of ~340 pS followed by an intermediate MscCG2 conductance of ~1.0 nS and comparably very large conductance of 3.7 nS exhibited by CgMscL. By applying Laplace’s law, we determined that very moderate membrane tension of ~5.5 mN/m was required for half activation of MscCG compared to ~12 mN/m required for half activation of both MscCG2 and CgMscL. Furthermore, by combining the micropipette aspiration technique with molecular dynamics simulations we measured mechanical properties of the *C. glutamicum* membrane, whose area elasticity module of *K*_A_ ≈ 15 mN/m is characteristic of a very soft membrane compared to the three times larger area expansion modulus of *K*_A_ ≈ 44 mN/m of the more elastic *E. coli* membrane. Moreover, we demonstrate that the “soft” properties of the *C. glutamicum* membrane have a significant impact on the MscCG gating characterized by a strong voltage-dependent hysteresis in the membrane of *C. glutamicum* compared to a complete absence of the hysteresis in the *E. coli* cell membrane. We thus propose that MscCG has evolved and adapted as an MscS-like channel to the mechanical properties of the *C. glutamicum* membrane enabling the channel to specialize in transport of amino acids such as glutamate, which are major osmolytes helping the bacterial cells survive extreme osmotic stress.

## Introduction

Bacterial cells possess two types of mechanosensitive channels, the MscS-like and the MscL-like channels^1^. These mechanosensitive channels serve in regulation of cellular turgor pressure in response to changes in environmental osmolarity^2^. For example, when *E. coli* cells are exposed to hypoosmotic shock, MscS is activated first at the moderate membrane tension, and then, MscL opens close to the membrane lytic tension to prevent the cells from bursting^3^. This osmoregulation system is evolutionarily highly adapted for bacterial survival upon the osmotic stress. *E. coli* possesses one MscL channel and six MscS-like channel proteins: MscS, MscK, YbdG, YnaI, YbiO, and YjeP^4^. These mechanosensitive channels have different activation thresholds and channel conductance allowing the bacterial cells to adapt to a wide range of changes in their osmotic environment. Moreover, MscS channel exhibits fast gating kinetics enabling it to respond quickly to osmotic environmental changes as well as a strong inactivation and desensitization mechanism to prevent the excess efflux of osmolytes^5,6^.

MscS and MscL sense the mechanical force directly from membrane lipids^7^. Consequently, their structural and functional diversity is thus of great interest to understand how different mechanosensitive channels have adapted to function in their membrane environment through their interaction with membrane lipids. Originally, MscS and MscL were discovered in patch clamp experiments from *E. coli* giant spheroplasts^8^ and were later shown to function as osmotic safety valves in the *E. coli* cell membrane^2^. However, recent progress in sequencing of very large number of bacterial genomes has revealed that MscS-like channels have evolved and adapted to membrane environments in a large variety of cell-walled organisms from bacteria to plants whilst they diversified their structure and function during their molecular evolution^9–14^. As biological molecules, the family of MscS-like channels provide an excellent example of evolutionary ‘tinkering’^15,16^, which has taken MscS-like channels from their physiological function in bacterial osmoregulation to roles in diverse processes, such as amino acid efflux, Ca^2+^ regulation, chloroplast organization, and cell division. A likely explanation of the diversity of the MscS family is a gene horizontal transfer^17^. The first ancestral MscS gene was most probably duplicated in the bacterial genome, and the divergence occurred to create multiple MscS paralogs. These paralogs might have undergone gene fusion providing them with specific functions different from the canonical MscS channel. For example, the MscS-like KefA of *E. coli*, known as MscK, is one of the MscS paralogs, which has multiple additional transmembrane helices at the N-terminus and exhibits K^+^-dependent mechanosensitive channel activities^18^.

*C. glutamicum* MscCG (first reported as NCgl1221^19,20^) channel is one of the MscS-like channels, which has evolved and specialized in the amino acid transport and has therefore, been used for many years in industrial production of large amounts of glutamate exported when membrane tension is increased by the treatment of *C. glutamicum* cells with penicillin, Tween-40, and biotin limitation^21^. Unlike in *E. coli*, mechanosensitive channels seem to play a lesser role for the survival in *C. glutamicum* upon hypoosmotic shock because of the very thick cell-wall protecting this bacterium similar to other mycobacteria^22^. However, the MscCG channel has been shown to function in the fine-tuning of the cytoplasmic solute concentration upon hyperosmotic stress as a passive, but regulated efflux channel in the “pump and leak” mechanism in cooperation with the active and regulated glutamate uptake via the BetP carrier^23^. MscCG has been identified as the major export system utilized industrially for production of monosodium glutamate (MSG) for more than half a century. The characteristic structural feature of MscCG is a large carboxy-terminal extended domain, which is absent in the canonical *E. coli* MscS^24^. Recently, another MscS-like channel, MscCG2, has been reported as a minor accessory glutamate exporter in a *C. glutamicum* strain^25^. This channel does not have the C-terminal extension found in MscCG, which was reported as being important for the glutamate export^26^. When MscCG is expressed heterologously in *E. coli* and its activity is recorded from the *E. coli* giant spheroplasts the channel, unlike MscS, exhibits slow gating kinetics and no inactivation or desensitization suggesting that these channels in addition to functioning as osmotic valves may also serve as metabolic valves^27^. MscCG, like MscS and MscL of *E. coli*, senses directly the “force-from-lipids” transferred to the channel through the cell membrane bilayer and activating it^28^. Expression of MscCG and MscCG2 gain-of-function (GOF) mutants causes a constitutive glutamate export in *C. glutamicum*^24,25,29^. The MscCG GOF mutants are activated at a lower membrane tension compared to the wild-type (WT) channel and exhibit a stronger gating hysteresis than the WT channel^30^. Given that *C. glutamicum* bacteria belong to mycobacteria and their membrane lipids consist almost completely of negatively charged lipids, including cardiolipin and phosphatidylglycerol, *C. glutamicum* channels “feel” the “force-from-lipids” in a very different membrane environment compared to the one characteristic of *E. coli*.

In this study, we have developed *C. glutamicum* giant spheroplasts amenable to the patch-clamp technique and demonstrate for the first time that endogenous mechanosensitive channel activities of MscCG, MscCG2, and CgMscL can all be recorded from this novel bacterial spheroplast preparation. Furthermore, we have also been able to determine that cell membrane of *C. glutamicum* is very soft and expandable, which may allow for a large cell volume change upon hypoosmotic shock. In addition, we also propose an explanation why only MscCG, and not MscCG2 and CgMscL, acts as a major glutamate exporter in *C. glutamicum* cells.

## Results

### Structural features and molecular evolution of MscS-like mechanosensitive channels in Corynebacterium species

**Figure 1:**
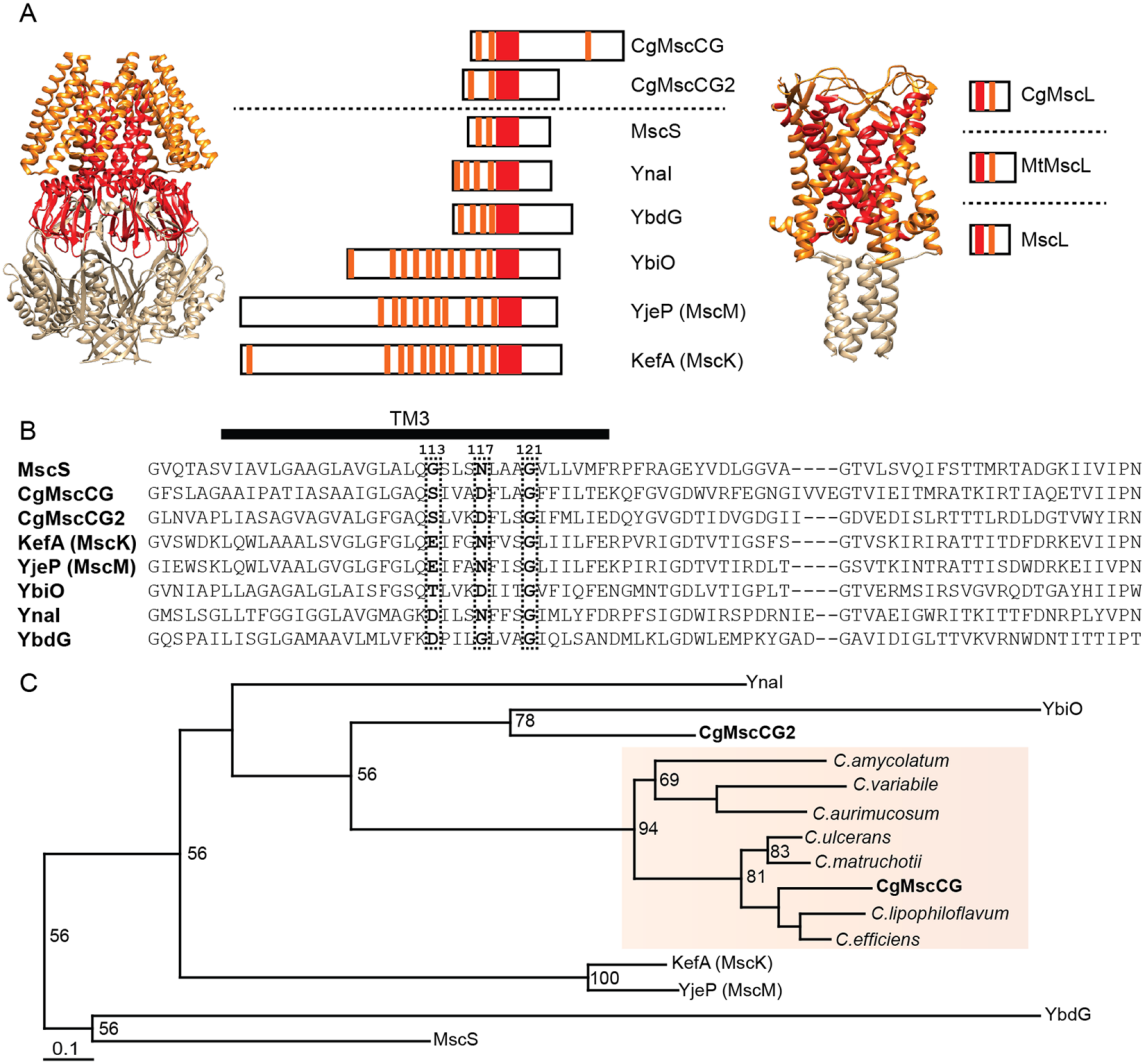
Mechanosensitive channels of *Corynebacterium glutamicum*. (**A**) Comparison of the two mechanosensitive channel genes MscS and MscL between *C. glutamicum* and *E. coli*. The crystal structure of the *E. coli* MscS heptamer (PDB: 2OAU)^50^ and the *M. tuberculosis* MscL pentamer (PDB: 2OAR)^51^ are shown on the left side. The predicted secondary structures of MscS homologs in *C. glutamicum* and *E. coli*, and the predicted secondary structures of MscL homologs in *C. glutamicum, M. tuberculosis*, and *E. coli* are shown on the right side, respectively. Orange bars indicate predicted transmembrane helices by TMHMM (http://www.cbs.dtu.dk/services/TMHMM-2.0/) and red bars indicate the MscS family conserved region including the pore-forming transmembrane helix. (**B**) Sequence alignment of the MscS family conserved region of *C. glutamicum* CgMscCG, CgMscCG2, and six MscS paralogs of *E. coli*. The black bar indicates the pore-lining transmembrane helix, and the two glycine residues G113 and G121 known as kink and the asparagine residue N117 important for the inactivation shown in MscS are highlighted. (**C**) A phylogenetic tree built by the maximum likelihood method using the MscS conserved domain. Orange box shows clustered CgMscCG-type channels. The number at the branches indicates bootstrap value larger than 50, out of 100 bootstraps. Note that the bootstrap values are only an indication of the node quality in the phylogenetic tree, but not for the phylogenetic tree shape. Also note that MscCG2 is located in the same branch with YbiO with the bootstrap score of 78.

*Corynebacterium glutamicum* possesses two MscS-like channels (MscCG and MscCG2)^19,25^ and one MscL-like channel CgMscL whereas *E. coli* has six MscS-like channels (MscS, KefA, YbdG, YbiO, YnaI, and YjeP) and one MscL channel. Note that the crystal structures of the *E. coli* MscS and *Mycobacterium tuberculosis* MscL channels were solved as a functional heptamer and pentamer, respectively (Fig. 1A). The primary amino acid sequence of the *C. glutamicum* MscCG (CgMscCG) has a large C-terminal extension (287–533 aa) not present in *E. coli* MscS. This C-terminal extension is characteristic of the MscCG-type channels found in *Corynebacteria* species (Supplementary Table S1). The third pore-lining transmembrane helix and the adjacent region known as the “MscS family conserved region” (shown in red in Fig. 1A) is also highly conserved in the MscCG channels, although several residues in this region important for the channel gating differ from the residues in MscS (Figs 1B, and S1A,B). In the MscCG-type channels the G113 residue important for the channel inactivation, is replaced by Ser^31^. Also, N117 residue, which is involved in the MscS inactivation^32^, is replaced by negatively charged Asp in *Corynebacteria* species. The characteristic fourth transmembrane helix at the C-terminal end is conserved as well in the MscCG channels across the species (Supplementary Fig. S1A,B).

Given that the MscCG channel functions as a glutamate exporter it is possible that MscCG-type channels have evolved differently from the canonical MscS channel. Note that the canonical MscS is the smallest protein in the MscS channel family, which does not necessarily mean that this channel is the ancestral channel. It is also possible that the ancestral channel was larger and that it had a more specialized function, whereas the current canonical MscS might be a structurally stripped-down version of a structurally larger MscS-like channel. To test this hypothesis, we performed phylogenetic analysis using the MscS conserved region. MscCG-type channels showed the best homology in this region among *Corynebacteria* species. Multiple sequence alignment revealed however, that MscCG (CgMscCG) and MscCG2 (CgMscCG2) are more similar to KefA (MscK), YjeP (MscM), YbiO, and YnaI, than to the canonical MscS (Figs 1B, S1A and Table S1). Consistently, a phylogenetic tree based on the maximum likelihood method supports the view that MscCG-type channels were grouped into a separate branch distant from the canonical MscS (Fig. 1C). Although 50 may not be a very good bootstrap score for most of the shown branches, MscCG2 is located in the same branch with YbiO with the bootstrap score of 78, which suggests that MscCG-type channels might have co-evolved with the YbiO channel, rather than evolved from the canonical MscS. Note that this result does not mean YbiO is the ancestral channel of MscCG and MscCG2. These results indicate that MscCG channels may have evolved as paralogs after the gene duplication of the MscS gene and acquired more specialized functions different from the common ancestral gene.

### Preparation of *C. glutamicum* giant spheroplasts

**Figure 2:**
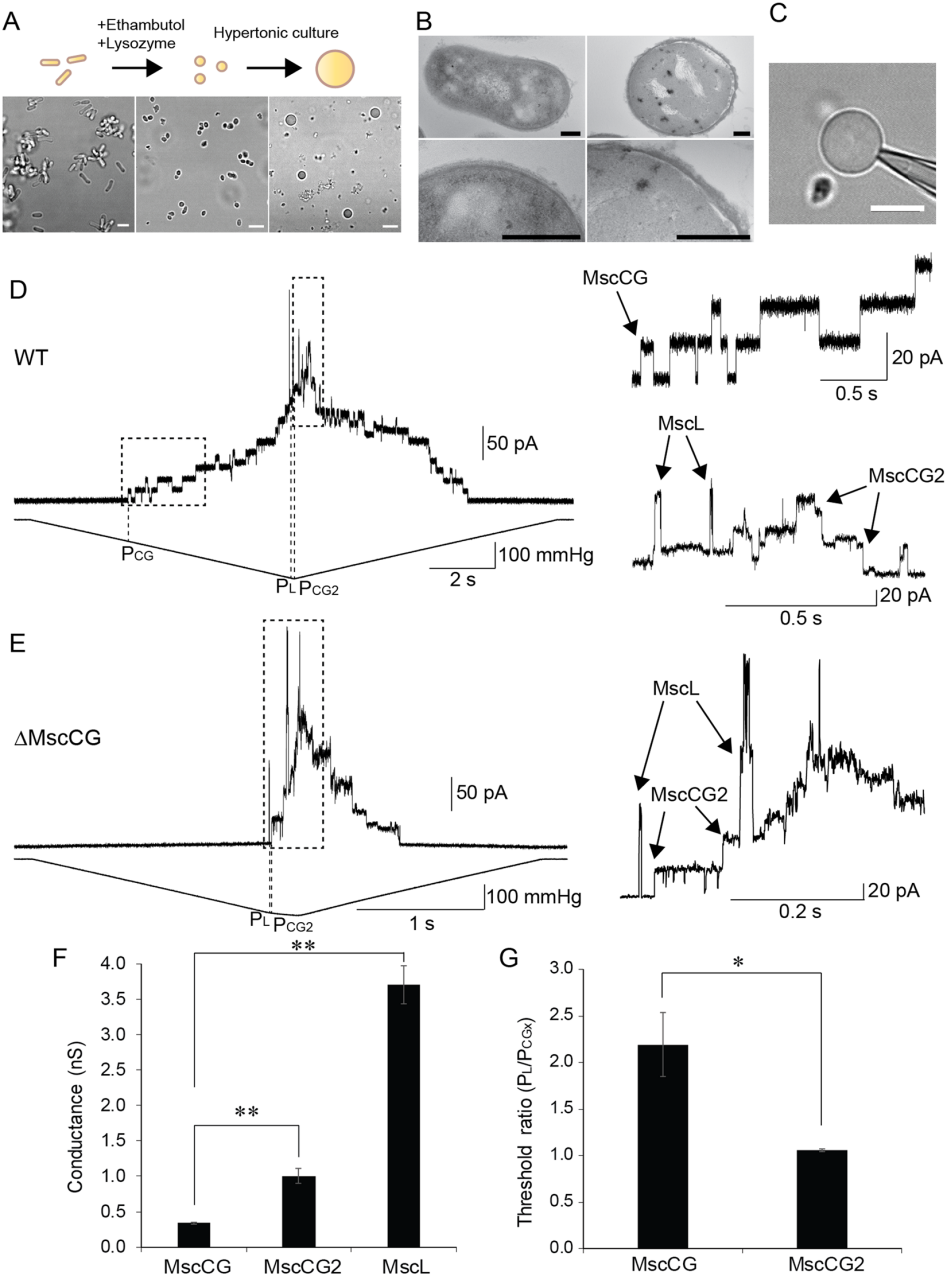
Mechanosensitive channel activities detected from *C. glutamicum* giant spheroplasts. (**A)** A diagram showing *C. glutamicum* giant spheroplasts preparation (top). Images of normal cells (left), round cells after treatment of ethambutol and lysozyme (center) and enlarged cells (right) (bottom). (**B)** Transmission electron microscopy of *C. glutamicum* giant spheroplasts. The shape of *C. glutamicum* normal cell (left). Image of a giant spheroplast (right). Magnified images of the cell wall are shown at the bottom. Scale bars correspond to 200 nm. (**C)** Image of cell-attached configuration of the *C. glutamicum* giant spheroplasts. Scale bar corresponds to 5 μm. (**D**) Three types of mechanosensitive currents in the *C. glutamicum* native membrane of WT strain at the pipette voltage of +60 mV. Channel current traces (top) and pressure traces applied to the membrane (bottom) in the inside-out excised patch configuration are shown. Magnified traces of CgMscCG (top), and CgMscCG2 and CgMscL (bottom) channels of the traces enclosed by dotted squares are shown. (**E**) Two types of mechanosensitive currents in the native membrane of *CgmscCG*-KO strain recorded at the pipette voltage of +30 mV. Magnified trace of CgMscCG2 and CgMscL channels from the traces enclosed by dotted squares is shown. (**F**) Single channel conductance of CgMscCG, CgMscCG2, and CgMscL. Channel currents were recorded in 200 mM KCl, 90 mM MgCl_2_, 10 mM CaCl_2_, 5 mM HEPES-KOH pH 7.0 at the pipette voltage of +30 mV. The channel conductance was calculated as the single channel current divided by the voltage. Bars show standard errors (n = 3). (*Student’s t-test, *P* < 0.05, **Student’s t-test, *P* < 0.01). (**G**) Activation thresholds of CgMscCG and CgMscCG2. The pressure required for the first activation of CgMscCG (P_CG_), CgMscCG2 (P_CG2_), and CgMscL (P_L_) was measured as shown in D and E, and the activation threshold ratio against P_L_ was calculated. Bars show standard errors (n = 5) (*student’s t-test, *P* < 0.05).

Similar to the preparations of giant spheroplasts in *E. coli*^8^, *Vibrio cholerae*^33^, and *Bacillus subtilis*^34^, lysozyme was also used in this study to digest the peptidoglycan layer during the preparation of giant spheroplasts in *C. glutamicum*. However, since *C. glutamicum* has an extremely thick cell-wall with an outer lipid-rich layer containing mycolic acids and arabinogalactan on top of the peptidoglycan layer, lysozyme alone was insufficient for digestion of the cell-wall. In addition to lysozyme we had to use ethambutol to remove the mycolic acids and arabinogalactan layer from *C. glutamicum* cells before treating the cells with lysozyme. *C. glutamicum* cells were grown in the peptone-yeast-glucose (PYG) medium. The cells became round after 21 h in the culture medium containing 2.5 mg/ml ethambutol. The peptidoglycan layer of the round cells was digested after 1 h upon addition of 10 mg/ml lysozyme, which also resulted in increased number of round cells demonstrating that the cell-wall could be digested by the combination of ethambutol and lysozyme (Fig. 2A).

The size of the round cell wall-less cells of ~1 μm in diameter was not large enough for the patch-clamp recording. To enlarge the cells further to ≥5 μm in diameter we let them grow for several days in the presence of ethambutol and ampicillin to inhibit the formation of the peptidoglycan layer and the synthesis of the arabinogalactan layer. Since the cells were osmotically very fragile, we cultured the cells in the presence of high concentration (0.8 M) of NaCl to prevent cell lysis. The cells grew gradually and their size plateaued at ~5 μm in diameter after 3 days (Supplementary Fig. S2A). We confirmed that these enlarged cells were *C. glutamicum* by 16S rRNA gene sequencing analysis (data not shown). When looking at the enlarged cells under a phase contrast microscope, we observed two types of cells having either black or white appearance, respectively (Supplementary Fig. S2B). To investigate whether the surface structure of the enlarged cells was clean enough to apply the patch-clamp technique, we examined them by transmission electron microscopy. As previously reported, we observed the thick cell-wall of *C. glutamicum* in the normal cell culture. Even though the enlarged cells had a different amount of the cell wall left, we were able to observe some enlarged cells with a relatively thin cell wall (Fig. 2B). Since some cell wall was still present in the enlarged cells, we named them “giant spheroplasts” in analogy to the giant spheroplasts from *E. coli* cells^8^.

### Mechanosensitive channels recorded in giant spheroplasts of *C. glutamicum*

To record mechanosensitive channel currents in the giant spheroplasts of *C. glutamicum* we first tested whether a tight gigaohm seal between the cell membrane and the glass wall of a patch pipette could be established. We attempted sealing on the giant spheroplasts of black appearance identified in the recording chamber with a phase-contrast microscope and were able to obtain a seal of ~1 GΩ (Fig. 2D). Moreover, we could excise the spheroplast membrane patch in the inside-out recording mode by snapping the pipette (Supplementary Video S1). In contrast, the spheroplasts of white appearance were difficult to seal on because their membrane was quite fluid and not amenable to form a gigaohm seal due to frequent vesiculation of the spheroplast membrane upon application of pipette suction (Supplementary Video S2). Consequently, we used only black giant spheroplasts for mechanosensitive channel recording in this study.

In order to detect mechanosensitive channel activities in the spheroplast membrane, we applied ramp pressure protocol that has been used in previous studies of *E. coli* MscS and MscL^1,35^. *E. coli* MscS and MscL were named as mechanosensitive channel of small conductance and mechanosensitive channel of large conductance, respectively, since these channels have significantly different conductance with 1 nS and 3 nS. Similarly, we were able to observe three different channel conductances in the *C. glutamicum* membrane upon the application of the ramp pressure. When the ramp pressure applied to the membrane gradually reached ~−100 mmHg at the pipette voltage of +30 mV, small currents of ~10 pA were observed first (Fig. 2D) and then after the saturation of these currents, larger currents of ~30 pA and ~100 pA were elicited at ~−210 mmHg (Fig. 2D). These three types of mechanosensitive channel activity ceased completely when the negative pressure was released (Fig. 2D). The smallest currents of ~10 pA are consistent with the conductance of MscCG reported previously^23,26,27^. To confirm the existence of MscCG in the spheroplast membrane, we recorded mechanosensitive currents in the giant spheroplasts of the MscCG-knockout strain. As expected, only two types of mechanosensitive channel activities of ~30 pA and ~100 pA corresponding to MscCG2 and CgMscL were observed at the pipette voltage of +30 mV (Fig. 2E), suggesting that the 10 pA small currents corresponded to MscCG. The 30 pA and 100 pA currents of MscCG2 and CgMscL, respectively, are comparable to that of *E. coli* MscS and MscL reported previously, and there is no other mechanosensitive gene found in the *C. glutamicum* genome. Note that MscCG currents were always elicited at the lowest pressure among the three types of mechanosensitive channels and did not show any sub-conductance states whereas the MscCG2 currents were elicited at almost the same level of pressure to activate CgMscL and exhibited frequent sub-conductance levels (Fig. 2D,E). From a number of experiments, we determined the average single channel conductance by calculating current amplitude divided by applied voltage. The calculated conductances of MscCG (n = 3), MscCG2 (n = 3), and CgMscL (n = 3), were 0.34 ± 0.01, 1.00 ± 0.11, and 3.70 ± 0.27 nS, respectively (Fig. 2F). This result shows that MscCG2 and CgMscL are comparable to the conductance of *E. coli* MscS (~1 nS) and MscL (~3 nS), and that these three channels can be identified by their conductances. To evaluate mechanosensitivity of MscCG, MscCG2, and CgMscL, we measured the threshold pressure required for the first channel opening observed in a recording (P_CG_, P_CG2,_ and P_L_) from which we calculated the activation threshold ratio of each channel relative to the CgMscL threshold. P_L_/P_CG_ was 2.19 ± 0.35 (n = 3), whereas P_L_/P_CG2_ was 1.06 ± 0.02 (n = 5) (Fig. 2G), indicating that MscCG2 requires much higher pressure for activation than MscCG given that its activation threshold is almost identical to MscL.

**Figure 3:**
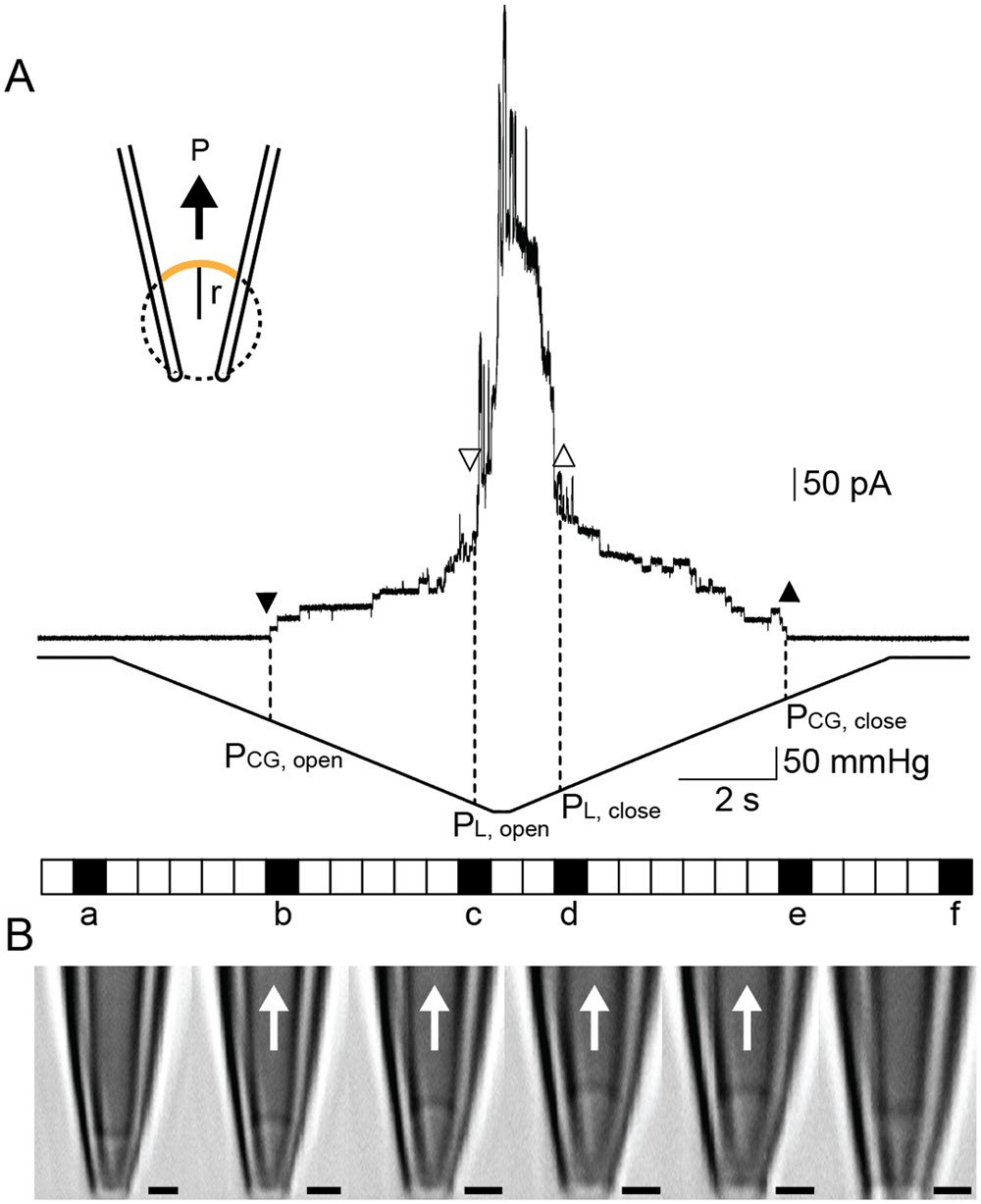
Determination of the gating thresholds of CgMscCG and CgMscL channels recorded in the *C. glutamicum* membrane. (**A**) A scheme of the membrane tension determination using the Laplace’s law (inset), CgMscCG and CgMscL channel currents in the inside-out configuration (top). Pressure applied to the membrane patch was controlled by a High-Speed Pressure Clamp (HSPC) apparatus. Dotted lines and arrow heads indicate the opening (downward) and closing (upward) of CgMscCG (closed) and CgMscL (open) channels. All images were taken during the 0.7 s time intervals shown as squares (bottom). (**B**) Images of the patch membrane from the intervals, a, b, c, d, e, f. Arrows show the direction of negative pressure. Scale bars correspond to 1 μm.

We further determined the membrane tension required to activate MscCG and CgMscL in the *C. glutamicum* native membrane since bacterial mechanosensitive channels have been shown to be activated by membrane tension, but not pressure^36^. To determine membrane tension in spheroplast patches we measured the radius of curvature (r) of the giant spheroplast membrane patch using a confocal microscope equipped with the patch-clamp system. To calculate membrane tension (T), we used the Laplace’s law (T = Pr/2)^37^, which relates membrane tension T to the applied negative pressure P and the radius of the patch curvature r (Fig. 3A). The amount of applied suction was measured as negative pressure (P) by a piezoelectric pressure transducer (Omega Engineering). Given the minimum recording interval time of 0.7 s in our confocal microscope system, which is much longer compared to the time resolution of the patch-clamp recording, we applied a slow pressure ramp to activate MS channels and recorded the images of the membrane patch at the exact time when channels started to gate first in order to determine the corresponding radius of the membrane curvature. MscCG was activated at ~−85 to ~−105 mmHg within the time interval b (Fig. 3A,B). At that time, the radius of the patch curvature was 0.9 μm, which corresponded to membrane tension between ~5.1 and ~6.3 mN/m. The CgMscL channels were activated at higher negative pressure of ~−226 to ~−240 mmHg within the time interval c when the radius of the patch curvature was 0.8 μm (Fig. 3A,B) corresponding to the threshold of activation by membrane tension between ~12.1 and ~12.8 mN/m. Note that although the radius of the membrane curvature did not change much at the activation points for MscCG and CgMscL in our experiments, the pressures required to activate each channel were very different. These values are comparable to the activation thresholds of MscS and MscL in azolectin liposome membrane, which were previously determined as ~6 mN/m and ~12 mN/m, respectively^37^. However, the activation threshold determined for MscCG in the *C. glutamicum* membrane (~5.1 to ~6.3 mN/m) is much smaller compared to the threshold of 9–20 mN/m required for MscS as previously determined in the *E. coli* giant spheroplast membrane^38^.

### Experimental determination of mechanical properties of the *C. glutamicum* and *E. coli* membranes

**Figure 4:**
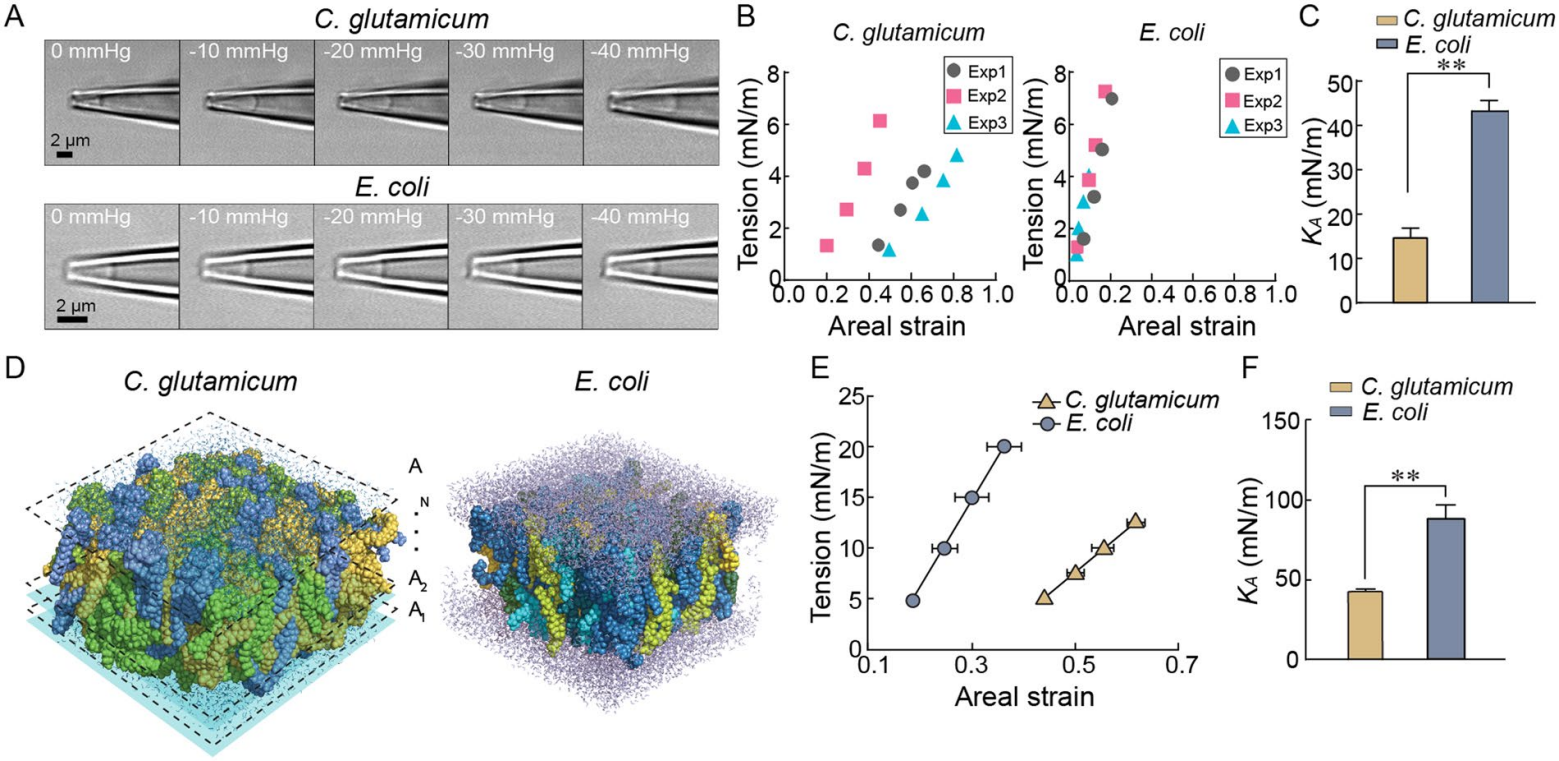
Mechanical properties of the *C. glutamicum* and *E. coli* membrane. (**A**) Representative images of how the *C. glutamicum* (top) and *E. coli* (bottom) membrane expand in response to the application of negative pressure (−10 mmHg steps), respectively. (**B**) The quasi-linear relationship between the membrane tension and the areal strain is shown for *C. glutamicum* (left) and *E. coli* (right). The slopes in these diagrams represent the areal elasticity modulus *K*_A_ for each membrane. (**C**) The comparison of *K*_A_ between the *C. glutamicum* and *E. coli* membrane. The experiments were performed more than three times independently. Bars show standard error (**student’s t-test, *P* < 0.01). (**D**) The solvated *C. glutamicum* lipid bilayer composed of their lipid components. POPG (yellow), POPI (blue), and cardiolipin (green) are shown, and the cross-sectional areas of the lipid along the bilayer thickness are shown as A_i_ (i = 1, 2, …, N) (left). The *E. coli* lipid bilayer composed of their lipid components (right). Four types of PE lipids (POPE, PMPE, QMPE, and OSPE) and two types of PG (PMPG and PSPG) are shown (See Supplemental Table S1). (**E**) The relationship between membrane tension and areal strain is shown for the *C. glutamicum* and *E. coli* lipid bilayers. (**F**) The comparison of *K*_A_ estimated by MD simulations between the *C. glutamicum* and *E. coli* membrane lipid bilayers. Three data points of independent simulations were analyzed, and bars represent SEM (**student’s t-test, *P* < 0.01).

MS channels sense the force from membrane lipids^7,28^, and thus, it is expected that the lipid composition and physical properties of the membrane bilayer would affect the mechanosensitivity of these channels. For example, activation thresholds of *E. coli* MscS and MscL differ significantly between azolectin liposomes and *E. coli* spheroplasts^33,34^. One of the explanations for this difference is that the areal elasticity modulus *K*_A_ of the *E. coli* spheroplast membrane is three times larger than the elasticity modulus of liposome membranes. The *C. glutamicum* membrane consists mainly of cardiolipin and phosphatidyl-glycerol, which is very different from the *E. coli* membrane consisting mainly of phosphatidyl-ethanolamine and phosphatidyl-glycerol^39^. We measured the mechanical properties of the *C. glutamicum* membrane using the micropipette aspiration (MA) method as previously done for the liposome membranes^40^. In the MA method, membranes are pulled into a pipette and the length of the pulled membrane inside the pipette (displacement) is measured after applying a constant negative pressure. Using a recently developed framework of patch-fluorometry for azolectin liposome membranes^40^, we were able to determine the areal elasticity modulus *K*_A_ of both the *C. glutamicum* and *E. coli* membranes. Both membranes were pulled into pipettes by applying −10, −20, −30, and −40 mmHg pressure steps (Fig. 4A, and Supplementary Videos S3 and S4). The *K*_A_ was calculated by plotting the relationship between the membrane tension and the areal strain (Fig. 4B). The *K*_A_ of the *C. glutamicum* membrane was calculated as 14.7 ± 2.2 mN/m (n = 3), whereas the *K*_A_ of the *E. coli* membrane was 43.7 ± 2.3 mN/m (n = 3) (Fig. 4C), suggesting that the *C. glutamicum* membrane is much softer than the *E. coli* membrane.

### MD simulations of mechanical properties of the *C. glutamicum* and *E. coli* membranes

Given that the *C. glutamicum* membrane consists of only negatively charged lipids, we employed MD simulations to examine whether the difference observed between the *C. glutamicum* and *E. coli* membranes stem from their membrane lipid composition. We modelled the *C. glutamicum* membrane based on its lipid composition previously reported^39^ according to which the lipid composition was 50.2% 1-palmitoyl-2-oleoyl-sn-glycero-3-phosphoglycerol (POPG), 30.2% 1-palmitoyl-2-oleoyl-sn-glycero-3-phosphoinositol (POPI), and 19.6%, cardiolipin (CL) molar ratio (Fig. 4D). The *E. coli* membrane model was created in parallel based on a previous study^41^, according to which the molar ratio of phosphatidyl-ethanolamine to phosphatidyl-glycerol was ~4:1. Both membrane bilayer models were stretched in MD simulations by surface tensions comparable to those applied in the MA experiments, i.e. 5, 10, 15, and 20 mN/m for the *E. coli* membrane and 5, 7.5, 10, and 12.5 mN/m for the *C. glutamicum* membrane. The results showed that the response of both membrane models to membrane tension was quasi-linear (Fig. 4E). The *K*_A_ was 42.1 ± 1.5 mN/m for *C. glutamicum* membrane and 87.5 ± 9.5 mN/m for *E. coli* membrane, respectively (Fig. 4F). Although individually larger than the experimentally determined values of *K*_A_ both values are comparable and of the same order of magnitude. These results suggest that the mechanical properties of the *C. glutamicum* membrane are comparable to those of liposome membranes rather than to the *E. coli* membrane. They also indicate that the membrane softness of *C. glutamicum* membrane originates from its lipid components.

### MscCG channels exhibit voltage-dependent gating hysteresis in *C. glutamicum* but not in *E. coli*

**Figure 5:**
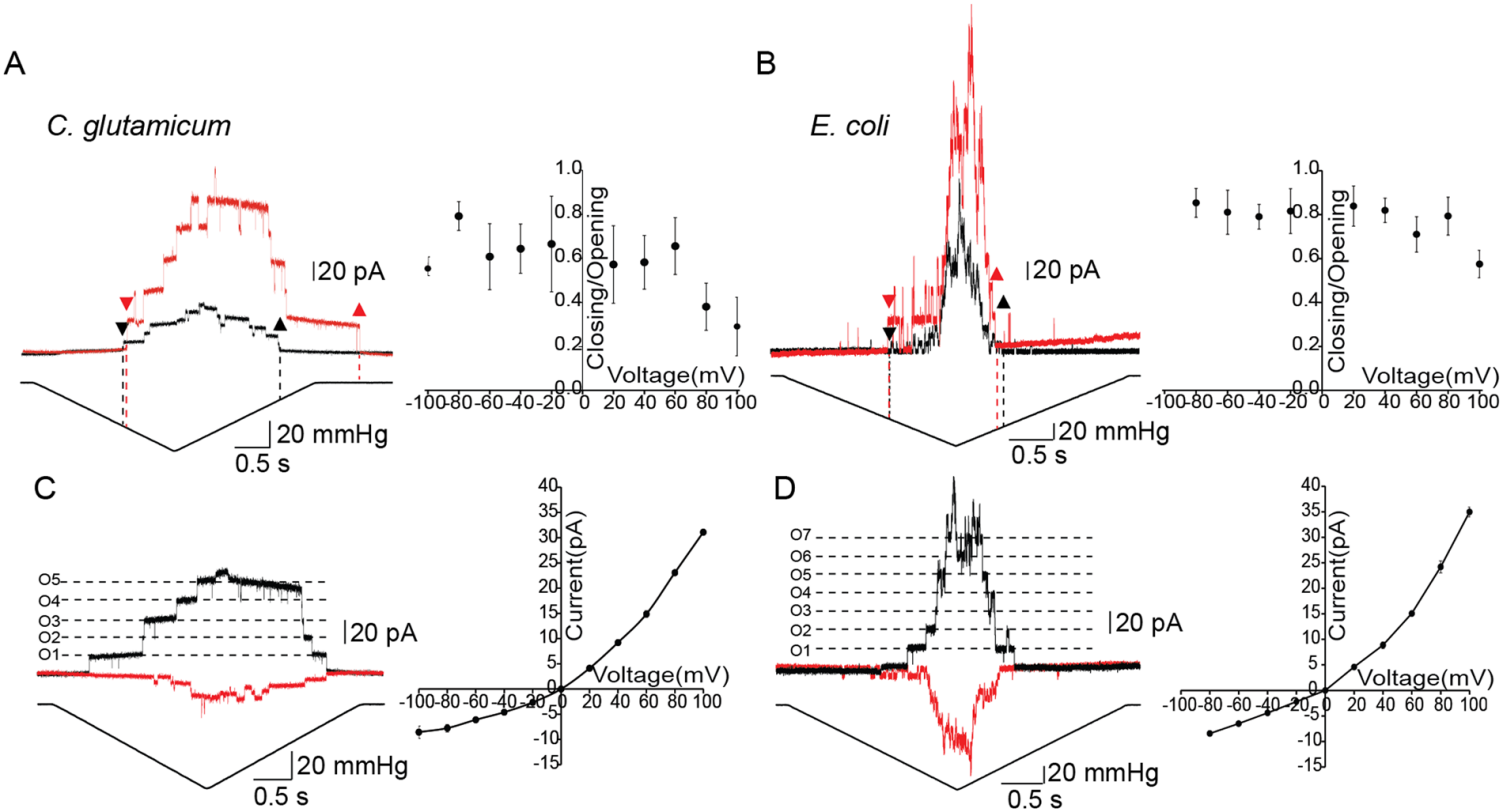
Gating characteristics of CgMscCG in the *C. glutamicum* and *E. coli* spheroplast membranes. (**A**) Current traces of CgMscCG in the *C. glutamicum* membranes at the pipette voltages of +40 mV (black) and +100 mV (red). Arrow heads indicate the opening (downward) and closing (upward) of CgMscCG. Gating hysteresis was determined by the gating threshold ratio between the first closing and opening pressure (dotted lines) and is plotted in the range of pipette voltages from −100 mV to +100 mV. Bars represent SEM (n = 4). (**B**) Gating hysteresis of CgMscCG in the *E. coli* membrane. Channel currents at the pipette voltages of +40 mV (black) and +100 mV (red) are shown on the left and voltage dependence of the gating threshold ratio is shown as in (A) on the right. Note that the pressure profile is different from (A) because of the strong gating hysteresis of CgMscCG in the *C. glutamicum* membrane. (**C**) Current rectification of CgMscCG in the *C. glutamicum* membrane. Current traces upon the application of a pressure ramp at the pipette voltages of +60 mV and −60 mV are shown in black and red, respectively (left). O1-5 indicates the number of open channels in the patch membrane. A current-voltage relationship of CgMscCG in the *C. glutamicum* membrane is shown on the right. Bars represent SEM (n = 6). (**D**) CgMscCG currents recorded in the *E. coli* membrane. O1-7 indicates the number of open channels in the membrane patch.

MscCG channels have been reported to function as “major glutamate exporters” in *C. glutamicum*, although interestingly, not as efficiently when expressed heterogeneously in *E. coli*^26^. The gating hysteresis defined as different gating thresholds between opening and closing has been suggested to be important for continuous glutamate export in *C. glutamicum*^26,27^. Thus, we examined to which extent the gating hysteresis of MscCG differs in the *C. glutamicum* and *E. coli* membranes. Since the MscCG currents can be distinguished from other currents of similar conductance, and MscCG is always activated at the lowest pressure among the three types of mechanosensitive channels, we were able to measure the single level of MscCG currents in the WT strain. We applied a slow ramp pressure that includes the same rate of loading and unloading to both membranes. MscCG opened at −34 mmHg and then closed at −14 mmHg in the *C. glutamicum* membrane whereas in the *E. coli* membrane, the opening and closing thresholds were −51 mmHg and −64 mmHg, respectively (Fig. 5A,B, black traces). Importantly, this gating hysteresis of MscCG was enhanced significantly only in the *C. glutamicum* membrane when a high pipette voltage was applied. At the pipette voltage of +100 mV (corresponding to the membrane potential of −100 mV in the inside-out patch recording mode) most of the MscCG channels remained open even when the pressure was completely released (Fig. 5A,B, red traces). To quantify the voltage effects on the gating hysteresis, we plotted the closing threshold/opening threshold ratio determined at pipette voltages from −100 mV to +100 mV (Fig. 5A,B). In the *C. glutamicum* membrane, the ratio became significantly decreased as the pipette voltage was increased. On the other hand, the gating hysteresis of MscCG did not change significantly and was even occasionally reversed in the *E. coli* membrane (Fig. 5B). These results suggest that the gating hysteresis observed with MscCG is voltage-dependent only in the *C. glutamicum* membrane. Note that the opening threshold of MscCG is unaffected by voltage, while the closing of MscCG channels slowed down dramatically at high positive pipette voltages resulting in the channels remaining open even upon release of suction applied to the patch membrane.

The current rectification of MscCG is also present in the *C. glutamicum* membrane as previously reported for MscCG recorded in *E. coli* membrane^23^. MscCG currents were significantly smaller (~70%) at negative pipette voltages compared to positive pipette voltages (Fig. 5C). This ~70% reduction was also observed in *E. coli* membrane (Fig. 5D). The current-voltage plots show the slope conductance of 286 pS and 95 pS in the *C. glutamicum* membrane at positive and negative pipette voltages, respectively, whereas the slope conductance in the *E. coli* membrane was larger at both voltages being 309 pS at positive and 107 pS at negative voltages (Fig, 5C,D).

## Discussion

We developed a *C. glutamicum* giant spheroplasts preparation amenable to the patch clamp technique and characterized for the first time the gating of endogenous mechanosensitive channels, MscCG, MscCG2, and CgMscL. Surprisingly, the electrophysiological characteristics of MscCG and MscCG2 were remarkably different, supporting the view that these two channels might have a different evolutionary origin as members of the MscS-like channel family. Given that most of the MscS-like channels have complex structure by having more than three transmembrane helices characteristic of the canonical MscS of *E. coli* and were shown to be involved in more specialized processes such as calcium signaling in fission yeast cellular signaling (Msy1 and Msy2)^9^ and apoptosis signaling in plants (MSL10)^42^, MscCG channels have four transmembrane helices and thus seem to have acquired more complex structure and gating characteristics for their more specialized function in *C. glutamicum*. Considering the differences in the gating and physiological function between MscCG and MscS it is likely that the MscCG channels have evolved after gene duplication of the *mscS* gene and “evolutionary tinkering” adding the fourth transmembrane helix and an extended C-terminal domain to enable its function as a glutamate exporter in *C. glutamicum*.

The electrophysiological characterization of MscCG functional properties in this study strongly supports the view for specialized function of MscCG in *C. glutamicum* cells. In addition to its low activation threshold of ~6 mN/m that is roughly half the activation threshold of CgMscL and MscCG2, MscCG lacks desensitization, inactivation and frequent sub-conducting states observed in the MscCG2 channels. All these characteristics are expected to contribute to a continuous export of glutamate as long as membrane tension keeps the channels open. Furthermore, MscCG exhibited a pronounced gating hysteresis in the *C. glutamicum* membrane characterized by a significant delay in the channel closing upon release of negative pressure applied to a spheroplast patch at low membrane potentials around −100 mV (corresponding to a pipette voltage of +100 mV in the inside-out patch recording mode). Since the resting membrane potential in a *C. glutamicum* cell is about −170 mV (corresponding to V_p_ = +170 mV in the inside-out excised patch configuration), MscCG may stay longer open at such high membrane potentials enabling more efficient export of glutamate facilitated by the absence of inactivation in MscCG due to mutations in residues shown to be important for the MscS channel inactivation^6^. Significantly, our data demonstrate the presence of strongly voltage-dependent gating hysteresis in MscCG, which was only observed with channels recorded in *C. glutamicum* giant spheroplasts and not with channels heterologously expressed in *E. coli* giant spheroplasts. This result indicates that although the hysteresis may be an inherent property of the channel protein itself as recently shown for the MscS channels^27,43^, in the MscCG case the hysteresis appears to be largely due to the properties of the negatively charged lipid membrane of *C. glutamicum*. How voltage may influence the closing of MscCG channels is not known. It is however, possible that the evolutionary modification through addition of a negatively charged loop connecting the TM3 and TM4 helices in the C-terminal domain may be the structural component of MscCG interacting electrostatically with the membrane. Given the importance of the C-terminal domain for the MscCG gating^26^, it is likely that the electrostatic interaction between the negative charges of the loop and the negatively charged membrane lipids is modulated by the cell membrane potential, which would affect the open probability and gating hysteresis of MscCG in a way promoting the glutamate export at high membrane potentials.

The *C. glutamicum* cytoplasmic membrane is made of negatively charged phosphatidyl-glycerol (PG), phosphatidyl-inositol (PI), and cardiolipin (CL). Our MA experiments and molecular dynamics simulations both indicate a mechanically very soft membrane. It has previously been shown that a lipid bilayer made of phosphatidyl-glycerol (PG) alone is much softer than a bilayer made of phosphatidyl-ethanolamine alone (PE)^41,44^, which suggests that the softness of the *C. glutamicum* membrane results from the negatively charged lipids. An interesting fact is that all mechanosensitive channels, MscCG, MscCG2, and CgMscL, are activated within a physiological range of membrane tension (≤20 mN/m) in this soft membrane. In *Mycobacterium tuberculosis*, the mechanosensitivity of MtMscL requires the presence of a small amount of PI to be activated within this physiological range of membrane tension, since it was shown that it is difficult to activate MtMscL when expressed in *E. coli* giant spheroplasts^45^. In support of this finding MtMscL was shown in mass-spectrometry experiments to associate with PI lipids^46^. The effects of the negatively charged lipids on the gating of MscCG thus require further study.

In summary, the gating of endogenous mechanosensitive channels, MscCG, MscCG2 and CgMscL found in *C. glutamicum* cytoplasmic membrane was characterized using the patch clamp recording of the channel activity from giant spheroplasts developed in this study. Our data strongly suggest that not only the properties of the MscCG channel protein but also its interactions with the lipid membrane environment have been evolutionarily fine-tuned towards the specialized function of this MscS-like channel as a glutamate exporter. Since *C. glutamicum* bacteria have been utilized for industrial glutamate production exceeding 2 billion tons per year^21^, the electrophysiological characterization of MscCG described here may in addition to contributing to a better understanding of the amino acid export mechanism facilitated by these channels also help to further improve the biotechnological process used industrially for amino acid production.

## Materials and Methods

### Strains, media and growth

The WT strain of *Corynebacterium glutamicum* ATCC13869 was used for all the experiments. The ΔMscCG strain of *Corynebacterium glutamicum* ATCC13869 generated for a different study^19^ was used in our study. Vegetable peptone (Oxoid, VEGETABLE PEPTONE No 1, Code: VG0100) was used for CM2B-V, PYG-V, and GP-V medium. After autoclaving, precipitation of the Vegetable peptone was observed, and all media were resuspended by shaking gently before the use. Cells were incubated at 31.5 °C, 135 rpm for aerobic culture with the incubator INNOVA 44 (Eppendorf, Germany) and collected with the centrifuge Sorvall Legend RT (Thermo scientific).

### Phylogenetic analysis

MscCG homologs were collected with the BLAST program (https://blast.ncbi.nlm.nih.gov/Blast.cgi) and candidates were screened based on the annotation. Multiple sequences were aligned with the CLUSTALW program (http://www.genome.jp/tools-bin/clustalw) as well as manually, and the sequences of the MscS conserved region were extracted to construct the phylogenetic tree with the TREE program (http://www.genome.jp/tools-bin/ete).

### Giant spheroplast preparation

*C. glutamicum* cells were inoculated on a CM2B-V agar plate (Vegetable peptone 10 g/L, Bacto Yeast Extract 10 g/L, NaCl 5 g/L, Agar 15 g/L, pH 7.0 adjusted with KOH, autoclaved at 121 °C, 15 min) and incubated at 31.5 °C overnight. The next day, cells were transferred using a pipette tip into 6.5 ml PYG-V medium (Vegetable peptone 5 g/L, Bacto Yeast Extract 2.5 g/L, Glucose 10 g/L, NaCl 0.1 g/L, CaCl_2_·2H_2_O 0.13 g/L, MgSO_4_·7H_2_O 0.3 g/L, MnSO_4_·H_2_O 0.014 g/L, FeSO_4_·7H_2_O 0.006 g/L, K_2_HPO_4_ 0.5 g/L, KH_2_PO_4_ 0.3 g/L, Biotin 30 μg/L, pH 6.8 adjusted with NaOH, autoclaved at 121 °C, 15 min) with 163 μl of 100 mg/ml ethambutol (final: 2.5 mg/ml) in a 100 ml flask, and then were incubated at 31.5 °C, 135 rpm for 21 h until reaching the late log-phase. Cell culture was transferred to another four 1.5 ml tubes and centrifuged at 2,500 *g*, 5 min, 4 °C. Supernatant was discarded, and all cell pellets were resuspended in 0.5 ml S. P. buffer (550 mM Sucrose, 25 mM Tris-HCl buffer, pH 6.8) in a tube. 100 μl of 50 mg/ml lysozyme was added and cells were incubated at 30 °C, 60 min to digest the cell wall. After the lysozyme treatment, 0.5 ml GP-V medium (Vegetable peptone 10 g/L, Bacto Yeast Extract 5 g/L, NaCl 20 g/L, CaCl_2_·2H_2_O 0.13 g/L, MgSO_4_·7H_2_O 0.3 g/L, MnSO_4_·H_2_O 0.014 g/L, FeSO_4_·7H_2_O 0.006 g/L, K_2_HPO_4_ 0.5 g/L, KH_2_PO_4_ 0.3 g/L, Biotin 30 μg/L, Thiamine-HCl 200 μg/L pH6.8 adjusted with NaOH, autoclaved at 121 °C, 15 min) was added and cell pellet was gently resuspended with a pipette. 65 μl of cell culture was transferred into 10.4 ml main medium (GP-V medium, 0.3 mg/ml Ampicillin, 2.5 mg/ml ethambutol) in 50 ml tube, and then incubated at 23 °C, 30 rpm to avoid aeration for about 2–4 days. 10.4 μl of 300 mg/ml Ampicillin and 260 μl of 100 mg/ml ethambutol were added every 24 and 48 hrs, respectively. Once spheroplasts became large enough for patch-clamp experiments cells were collected by centrifugation at 2,500 × *g* for 5 min at 4 °C. Supernatant was discarded and cell pellets were resuspended in 1 ml Wash buffer (800 mM Sucrose, 10 mM MgCl_2_, 10 mM Tris-HCl buffer, pH 7.2) in 1.5 ml tube. The aliquots of the cell suspension were stored at −80 °C.

### Transmission electron microscopy

Prefixation of *C. glutamicum* samples was performed in 2% Glutaraldehyde in 0.1 M phosphate buffer at 4 °C, and the samples were washed in 0.1 M phosphate buffer for overnight at 4 °C. Subsequently, postfixation was performed in 2% osmium tetroxide for 3 hours in an ice bath. Then, the specimens were dehydrated in a graded ethanol (30, 50, 70, 90, 100, 100, 100%) for 15 minutes each and incubated in the Gelatin capsule with epoxy resin for 2 days at 60 °C. 70–80 nm ultrathin sections with diamond knives were obtained using ultramicrotome technique. Ultrathin sections were mounted on 200 mesh copper grids and treated with 2% uranyl acetate in DW for 10 minutes and a lead staining solution for 5 minutes. The samples were observed with a TEM (HITACHI; H-7600).

### Patch-clamp recording

Glass pipettes (Drummond Scientific, Broomall, PA) were pulled with a Narishige gravity puller (PP-83; Narishige, Tokyo, Japan) to make pipettes of the bubble number of 4.0–6.0. Patch pipette solution (200 mM KCl, 40 mM MgCl_2_, 5 mM Hepes-KOH, pH 7.2) and bath solution (patch pipette solution supplemented with 300 mM sucrose) were used in all recordings. Pipette resistance was 3.0–5.0 MΩ in the patch-clamp recording solution. Currents were amplified with an Axopatch 200B amplifier (Axon Instruments), and data were acquired at a sampling rate of 5 kHz with 2 kHz filtration. Pressure ramp activating the channels was applied to patch pipettes using a high-pressure clamp apparatus (ALA Scientific Instruments, USA; HSPC-1) and was monitored using a piezoelectric pressure transducer (Omega Engineering). The channel currents were analysed using pClamp 10 analysis software (Molecular Devices, Sunnyvale, CA). To obtain a better seal by improving salt bridge between the patch membrane and the pipette glass wall, magnesium and calcium concentration was increased. The open probability from all channels was obtained by the single-channel analysis using Clampfit software.

### Micropipette aspiration method

The images of the spheroplast patch membrane of *C. glutamicum* and *E. coli* were observed with Zeiss LSM 700 confocal microscope using a long working distance water immersion objective (63×; NA 1.15; Carl Zeiss) in a Faraday cage. To visualize the membrane, the pipette tip was bent ~30 °C with a microforge (Narishige; MF-900) to make it parallel to the bottom of the chamber. The areal elasticity modulus *K*_A_ was determined from the slope of the membrane tension, vs. areal strain graph using the following equation:

1 $${K}_{A}={\rm{\Delta }}T/\alpha $$

Membrane tension, T, was calculated based on the principle of surface chemistry (Laplace’s law) and is expressed as follows,

2 $$T=\frac{P{R}_{d}}{2}$$

where R_d_ is the radius of curvature of the membrane patch. The areal strain (fractional area change) is conventionally calculated by measuring the relative change in the apparent area of the expanded membrane inside the pipette, A (after the application of suction), with respect to its initial area, A_0_ (zero pressure). The areal strain is thus defined by

3 $$\alpha =\frac{A-{A}_{0}}{{A}_{0}}$$

Using geometric relations, the area after the application of suction (A) was calculated depending on the shape of the pipette. If the pipette shape is conical like in our experiments, then the membrane surface area is calculated as

4 $$\pi (r+R)+\sqrt{{(R-r)}^{2}+{L}^{2}}+2\pi Rh$$

where L is the protrusion length, L; r and R are the pipette radii at the pipette tip and at the dome, respectively. h is the height of the dome of the patch.

### Molecular dynamics simulations

All MD simulations were performed with NAMD2.10^47^. Visual Molecular Dynamics (VMD)^48^ and Pymol (Molecular Graphics System, Version 1.8 Schrödinger, LLC) were used for all visualizations. CHARMM-GUI was used to generate the *C. glutamicum* membrane lipid system^49^. We used a model of top 6 lipid types found in the *E. coli* cell membrane as previously modelled^41^. The *C. glutamicum* membrane was modelled based on its main membrane lipid composition^39^. The lipid was solvated by adding 20 Å water boxes on the top and bottom of the membrane along the thickness direction. The TIP3P water molecule model was used along with the SOLVATE program (https://www.mpibpc.mpg.de/grubmueller/solvate). The lipid heads and tails were, in turn, randomized for ~1 ns at 298 K, while the rest of the system was fixed in an NVT system. Then the whole system was equilibrated for 30 ns with a time step of 1 fs with no restraints in an NPT ensemble. To gain the areal elasticity modulus *K*_A_ of the *C. glutamicum* and *E. coli* membrane lipid bilayers, they were both stretched with the surface tensions close to their experimental range in different sets of simulations for 25 ns with a time step of 1 fs and in NPT ensembles. The *E. coli* bilayer was simulated under the surface tensions of 5, 10, 15, and 20 mN/m. The *C. glutamicum* bilayer was, however, simulated under the surface tensions of 5, 7.5, 10, and 12.5 mN/m due to its lower lytic tension observed in the MA experiments. Each simulation was repeated three times for both *C. glutamicum* and *E. coli* membranes. In all the simulations, the particle-mesh Ewald (PME) method was used for computing electrostatic interactions beyond a real-space cut-off of 1.2 nm with a Fourier grid spacing of 0.1 nm. A modified Nosé-Hoover Langevin piston pressure control provided in NAMD was utilized to control fluctuations in the barostat (at 1 atm). This method is combined with a method of temperature control (at 298 K) (Langevin dynamics) to simulate the NPT ensemble. The CHARMM36 (C36) Force field was used for all MD calculations. For data analysis, a TCL code was developed and used to calculate the average surface area of the lipid through different trajectories in different simulations. This code was employed to average the area over N (e.g., 10) slabs along the bilayer thickness direction (between the phosphate groups) as the lipid area is not uniform across the thickness direction. Then an average of areas over different trajectories is calculated as follows,

$${\rm{A}}=\frac{{\sum }_{1}^{M}{\sum }_{1}^{N}{A}_{i}}{M\times N}$$

*M* is the number of steady trajectories. A_i_ (i = 1, 2, …, N) is the cross-sectional area of the lipid along the bilayer thickness (Fig. 4D). When the bilayer is stretched, the first few trajectories can be excluded from the deformed area analysis, to make sure the steady surface area is measured. The *K*_A_, was calculated using Eqns 1 and 2 for analyzing the micropipette aspiration data. To be able to obtain a statistically significant result the *K*_A_ values of the two models were compared and each four data points (e.g. at surface tensions of 5, 10, 15, 20 mN/m) were assumed as one data set.

## Electronic supplementary material

Video S1

Video S2

Video S3

Video S4

Supplementary Information
