# Supplementary material for: Evolutionary specialization of MscCG, an MscS-like mechanosensitive channel, in amino acid transport in Corynebacterium glutamicum
Source: Sci Rep. 2018 Aug 27;8:12893. doi: 10.1038/s41598-018-31219-6 (PMC6110860; doi:10.1038/s41598-018-31219-6)
Supplement: Supplementary file 5 — Supplementary Information [file 41598_2018_31219_MOESM5_ESM.docx]

**Supplementary information**

**Evolutionary specialization of MscCG, an MscS-like mechanosensitive channel, in amino acid transport in *Corynebacterium glutamicum***

Yoshitaka Nakayama ^a^, Kosuke Komazawa ^b^, Navid Bavi ^a,c^, Ken-ichi Hashimoto ^b^, Hisashi Kawasaki ^b^, Boris Martinac ^a,c, *^

1. Molecular Cardiology and Biophysics Division, Victor Chang Cardiac Research Institute, Darlinghurst, NSW 2010, Australia
2. Department of Green and Sustainable Chemistry, Tokyo Denki University, 5 Asahi-cho, Senju, Adachi-ku, Tokyo 120-8551, Japan
3. St Vincent’s Clinical School, Faculty of Medicine, University of New South Wales, Darlinghurst, NSW 2010, Australia

* Corresponding author: Boris Martinac

Victor Chang Cardiac Research Institute

405 Liverpool St, Darlinghurst, NSW 2010, Australia

Tel.: +61 2 9295 8743

Fax: +61 2 9295 8770

E-mail address: [b.martinac@victorchang.edu.au](mailto:b.martinac@victorchang.edu.au)

**
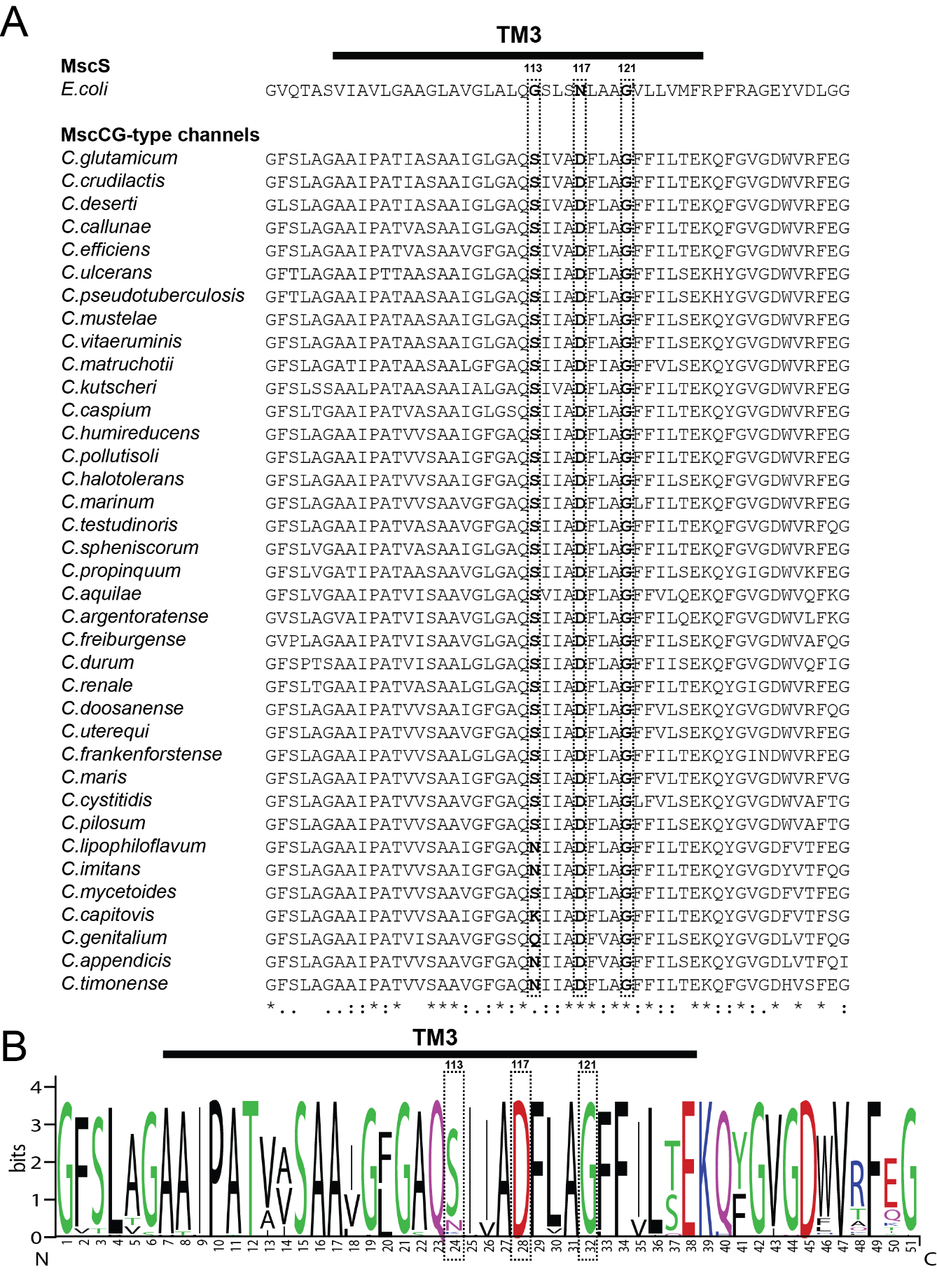
Figure S1** The conserved region of MscCG-type channels in *Corynebacteria* species. **A).** Sequence alignment of the pore-forming third transmembrane helix and adjacent region between *E. coli* MscS and MscCG-type channels in *Corynebacteria* species. The G113, G121, and N117 residues known as important for the inactivation in *E. coli* MscS are highlighted. **B).** The WebLogo analysis (http://weblogo.berkeley.edu/logo.cgi) of MscCG-type channels in *Corynebacteria* species in the conserved region.

**
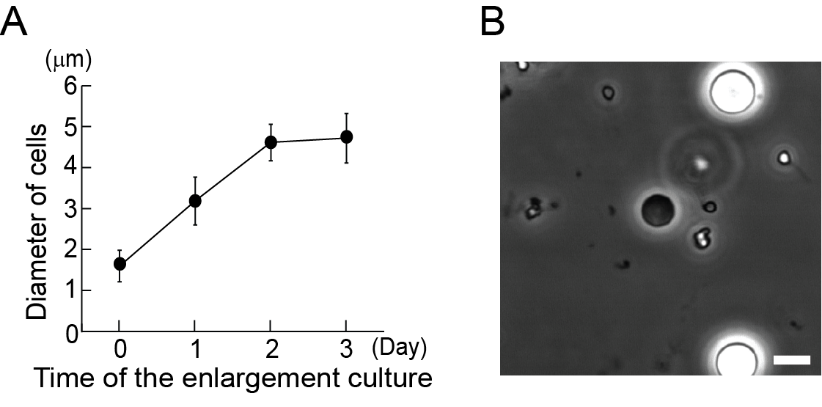
**

**Figure S2** Preparation of *C. glutamicum* giant spheroplasts. **A).** Time course of the cell size during the enlargement culture. Bars show standard error (n = 10). **B).** Two types of *C. glutamicum* giant spheroplasts, white and black cells, under a phase contrast microscope. Scale bars show 5 μm.

**Supplementary Table S1** List of MscCG-type channels and MscS paralogs used for phylogenetic analysis.

| Accession number | Species | Gene name | Size (a.a.) |
| --- | --- | --- | --- |
| BAE76988 | *Escherichia coli* | *YggB* (*MscS*) | 286 |
| CDZ19328 | *Escherichia coli* | *KefA* (*MscK*) | 1120 |
| BAE78163 | *Escherichia coli* | *YjeP* (*MscM*) | 1107 |
| BAA35474 | *Escherichia coli* | *YbiO* | 741 |
| BAA14923 | *Escherichia coli* | *YnaI* | 343 |
| BAA35217 | *Escherichia coli* | *YbdG* | 415 |
| NP_600492 | *Corynebacterium glutamicum* | *MscCG* | 533 |
| ANU34376 | *Corynebacterium glutamicum* | *MscCG2* | 334 |
| WP_066565323 | *Corynebacterium crudilactis* | *MscCG* | 521 |
| WP_053544706 | *Corynebacterium deserti* | *MscCG* | 545 |
| WP_015651054 | *Corynebacterium callunae* | *MscCG* | 537 |
| WP_006769328 | *Corynebacterium efficiens* | *MscCG* | 591 |
| KPJ24640 | *Corynebacterium ulcerans* | *MscCG* | 564 |
| AKP08551 | *Corynebacterium pseudotuberculosis* | *MscCG* | 564 |
| AKK05731 | *Corynebacterium mustelae* | *MscCG* | 510 |
| AHI22606 | *Corynebacterium vitaeruminis* | *MscCG* | 524 |
| WP_005523077 | *Corynebacterium matruchotii* | *MscCG* | 633 |
| AKE41033 | *Corynebacterium kutscheri* | *MscCG* | 548 |
| WP_083900479 | *Corynebacterium caspium* | *MscCG* | 546 |
| WP_040087376 | *Corynebacterium humireducens* | *MscCG* | 509 |
| SMG27975 | *Corynebacterium pollutisoli* | *MscCG* | 523 |
| WP_015400717 | *Corynebacterium halotolerans* | *MscCG* | 569 |
| WP_084603077 | *Corynebacterium marinum* | *MscCG* | 510 |
| AKK08570 | *Corynebacterium testudinoris* | *MscCG* | 478 |
| WP_092285395 | *Corynebacterium spheniscorum* | *MscCG* | 518 |
| WP_053083633 | *Corynebacterium propinquum* | *MscCG* | 412 |
| WP_075725884 | *Corynebacterium aquilae* | *MscCG* | 562 |
| WP_020976103 | *Corynebacterium argentoratense* | *MscCG* | 570 |
| WP_035112483 | *Corynebacterium freiburgense* | *MscCG* | 504 |
| EKX92127 | *Corynebacterium durum* | *MscCG* | 569 |
| PFG28871 | *Corynebacterium renale* | *MscCG* | 491 |
| AIT60915 | *Corynebacterium doosanense* | *MscCG* | 506 |
| AKK10948 | *Corynebacterium uterequi* | *MscCG* | 470 |
| WP_083666879 | *Corynebacterium frankenforstense* | *MscCG* | 614 |
| WP_020934610 | *Corynebacterium maris* | *MscCG* | 612 |
| SNV77585 | *Corynebacterium cystitidis* | *MscCG* | 545 |
| WP_018580794 | *Corynebacterium pilosum* | *MscCG* | 549 |
| WP_006840852 | *Corynebacterium lipophiloflavum* | *MscCG* | 478 |
| AIJ33390 | *Corynebacterium imitans* | *MscCG* | 459 |
| WP_092148053 | *Corynebacterium mycetoides* | *MscCG* | 491 |
| WP_018017795 | *Corynebacterium capitovis* | *MscCG* | 496 |
| WP_005290437 | *Corynebacterium genitalium* | *MscCG* | 492 |
| SIS40633 | *Corynebacterium appendicis* | *MscCG* | 541 |
| SDS16411 | *Corynebacterium timonense* | *MscCG* | 557 |

**Supplementary Table S2** List of *C. glutamicum* and *E. coli* membrane lipid components.

| *C. glutamicum* membrane model | | | |
| --- | --- | --- | --- |
| **Abbreviation** | **Full name** | **Representative colour** | |
| POPG | 1-palmitoyl-2-oleoyl-sn-glycero-3-phosphoglycerol | 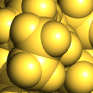 | ­­Yellow |
| POPI | 1-palmitoyl-2-oleoyl-sn-glycero-3-phosphoinositol | 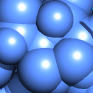 | Blue |
| CL | Cardiolipin | 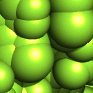 | Green |
| *E. coli* membrane model ^1^ | | | |
| **Abbreviation** | **Full name** | **Representative colour** | |
| POPE | 1-palmitoyl-2-oleoyl-sn-glycero-3-phosphoethanolamine | 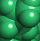 | Dark green |
| PMPE | 1-palmitoyl-2-cis-9,10-methylene-hexadecanoic-acid-sn-glycero-3-phosphoethanolamine | 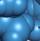 | Blue |
| QMPE | 1-pentadecanoyl-2-cis-9,10- methylene-hexadecanoic-acid-snglycero-3-phosphoethanolamine | 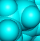 | Cyan |
| OSPE | 1-oleoyl-2-palmitoleoyl-snglycero-3-phosphoethanolamine | 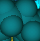 | Blue-green |
| PMPG | 1-palmitoyl-2-cis-9,10- methylene-hexadecanoic-acidglycero-sn-3-phosphoglycerol | 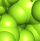 | Yellow-green |
| PSPG | 1-palmitoyl-2-palmitoleoyl-snglycero-3-phosphoglycerol | 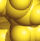 | Yellow |

**References**

1 Pandit, K. R. & Klauda, J. B. Membrane models of E. coli containing cyclic moieties in the aliphatic lipid chain. *Biochim Biophys Acta* **1818**, 1205-1210, doi:10.1016/j.bbamem.2012.01.009 (2012).

**Supplementary Video S1**

Making an inside-out excised patch configuration from a black *C. glutamicum* giant spheroplast.

**Supplementary Video S2**

Patching a white *C. glutamicum* giant spheroplast.

**Supplementary Video S3**

Applying the constant pressure steps -10, -20, -30, and -40 mmHg to the *C. glutamicum* patch membrane.

**Supplementary Video S4**

Applying the constant pressure steps -10, -20, -30, and -40 mmHg to the *E. coli* patch membrane.
